# Atropine and Scopolamine in Maize Products from the Retail Stores in the Republic of Serbia

**DOI:** 10.3390/toxins14090621

**Published:** 2022-09-05

**Authors:** Gorica Vuković, Tijana Stojanović, Bojan Konstantinović, Vojislava Bursić, Nikola Puvača, Milena Popov, Nataša Samardžić, Aleksandra Petrović, Dušan Marinković, Svetlana Roljević Nikolić, Rada Đurović Pejčev, Bojana Špirović Trifunović

**Affiliations:** 1 Faculty of Agriculture, University of Belgrade, Nemanjina 6, 11080 Belgrade, Serbia; 2 Department for Phytomedicine and Environmental Protection, Faculty of Agriculture, University of Novi Sad, Trg Dositeja Obradovića 8, 21000 Novi Sad, Serbia; 3 Department of Engineering Management in Biotechnology, Faculty of Economics and Engineering Management in Novi Sad, University Business Academy in Novi Sad, Cvećarska 2, 21000 Novi Sad, Serbia; 4 PSS Institute Tamiš Pančevo, Research and Experimental Development in Biotechnology, Novoseljanski put 33, 26000 Pancevo, Serbia; 5 Laboratory of Chemistry, Institute of Pesticides and Environmental Protection, Banatska 31b, 11080 Belgrade, Serbia

**Keywords:** toxins, atropine, scopolamine, LC-MS/MS, food safety

## Abstract

The cereal grains, which represent the cultivated grasses fruits, supply almost half of the total caloric requirements for humans and provide more nourishment compared with any other class of the food. Out of many cereals used for food, maize, rice, and wheat are the most important food resources for humans, representing 94% of the total cereals consumption. According to the data of the Republic Institute of Statistics for the year 2018, the harvested areas of corn amount to 906,753 hectares. The production of about 7 million tons was achieved with an average yield of 7.7 t/ha according to the Ministry of Agriculture of the Republic of Serbia. Serbia is still among the ten largest exporters of wheat and corn in the world for the period of 2014/15–2017/18. More precisely, it ranks seventh in the export of corn. Utilization of maize products for food animal nutrition (1000 t) is 491,48, and for industrial processing (1000 t) 278,862 expressed as the total consumption (1000 t) is 769,910. Therefore, a total of 103 samples of maize products were analyzed for the presence of toxins, i.e., tropane alkaloids (TAs). The samples were collected from the retail stores in the Republic of Serbia in 2021 and analyzed for the presence of atropine and scopolamine (33 corn grits, 39 polenta, and 31 semolina samples). Therefore, the Recommendation 2015/976/EU on the monitoring of TAs in food was adopted by the EU Commission to obtain more occurrence data on TAs in food. The monitoring extent, however, is restricted because reliable analytical methods and appropriate sensitivity are limited. There was a limit of 1 g/kg for each atropine and scopolamine in cereals containing millet, sorghum, buckwheat, or their derivatives. All the samples were analyzed by the LC-MS/MS. The LOQ was set at 1.0 μg/kg. Out of the total 103 tested samples, 32 samples (31.1%) were contaminated with atropine and scopolamine in concentrations above the LOQ. The highest concentrations of the studied TAs were observed in a semolina sample-atropine: 58.80 μg/kg, scopolamine: 10.20 μg/kg. The obtained results indicate that the TAs concentrations are above the LOQ which can be considered potential human and animal health hazards.

## 1. Introduction

The alkaloids, which comprise more than 27 thousand described structures, are a group of plants’ secondary metabolites which contains nitrogen in their structure [1,2]. According to the ring structures and biosynthetic pathways, several groups of alkaloids can be distinguished: indol, indolizidine, isoquinoline, monoterpene indole, piperidine, pyridine, pyrimidine, pyrrole, pyrrolizidine, quinoline, quinolizidine, steroidal, and terpenoid alkaloids [3,4]. In addition to carbon, hydrogen, and nitrogen, alkaloids can also contain chlorine, phosphorus, sulfur, and, rarely, bromine [5]. The alkaloids are produced by animals [6], bacteria [7], fungi [8], as well as plants [9]. Carl F. W. Meissner was the first to mention the word *alkaloid*, which was derived from the Arabic word *al-qali* relating to the plant as the first source of soda [10]. By using the fruits, leaves, roots, seeds, and stems as poisons and remedies, civilizations have been using alkaloids since ancient times [11]. Now, alkaloids are, among others, known as anesthetics, antibacterials, anticancer drugs, antihypertensive agents, antimalarials, spasmolysis agents, vasodilators, etc., [12]. Many wild and cultural plant species produce secondary biomolecules in concentrations that can have adverse effects on human and animal health when ingested. Various alkaloid groups, along with the tropane alkaloids, are, without a doubt, among the secondary biomolecules which can pose a serious threat to human health [13].

Tropane alkaloids (TAs) represent a group of more than 200 secondary metabolites that can be found in all parts of the many plant species from the *Brassicaceae*, *Convolvulaceae*, *Erythroxylaceae*, *Euphorbiaceae*, *Proteaceae*, and, mainly, the *Solanaceae* families [14]. The hazardous effects of the TAs on human health are associated with the muscarinic acetylcholine receptors inhibition in the autonomic as well as the central nervous system [15]. TAs can lead to many antimuscarinic effects, such as cardiac dysfunction, mydriasis and accommodation paralysis, inhibition of micturition, decrease in salivary secretion [16,17,18], etc. However, some TAs do not have that kind of effect due to their inability to pass the blood–brain barrier, such as calystegines. Some of the known TAs are atropine, convolamine, convolvine, hyoscyamine, littorrine, meteloidine, scopolamine, and tigloidine [3]. TAs are mostly the mono-, di-, and trihydroxypropane esters with a variety of hydroxylated arrangements, derived from acetoacetate and ornithine, with the pyrrolines being their precursors [10]. One of the main sources of contamination with the TAs is the TAs containing weeds which are being harvested along with the cultural crops [19,20,21]. In addition to their use as secondary plant metabolites, TAs have a long history of being used for herbal medicine, folk remedies, pain relief, and religious beliefs [22]. In plants, the levels of TAs vary between organs and are not uniform. Plant seeds, roots, and flowers contain the most amounts of (-)-hyoscyamine and (-)-scopolamine [13]. Unintentional consumption of jimsonweed can occur when the weed is accidentally mixed or blended with crops due to its invasiveness. Some reported cases of human poisoning due to contamination or mislabeling have been reported [13]. These cases have been associated with teas, honey, flour, beans, and vegetable bean salads. TAs are not known to be stable during food processing due to a lack of data. The boiling process of buckwheat flour results in the degradation of both atropine and scopolamine [23]. The degradation of TAs may be uncertain due to a matrix effect; buckwheat/millet samples spiked with jimsonweed seeds showed different degradation rates [24]. There is considerable emphasis on the presence of TAs in jimsonweed; however, low levels of TAs occur naturally in many other foods, including tomato, broccoli, potato, paprika, and other vegetables [25]. It has been noted that jimsonweed can be harvested accidentally alongside food crops that are meant for humans. This plant has caused poisoning incidents in a variety of food commodities and in a number of countries due to its presence in these products. Jimsonweed seed contamination has been reported in buckwheat in Europe [26], carrot and beans in Europe [13,27], and wheat-based bread in East Africa [28]. In spite of the fact that these contamination reports have been linked to the presence of the seeds, other poisoning incidents have been attributed to the presence of other plant parts, such as the leaves [29]. 

It is possible that the contamination of food could be due to the fact that the TAs are naturally present in the raw food material. As an alternative, contamination could result from the coharvesting of plants containing tropane alkaloids, such as jimsonweed (*Datura stramonium*), which belongs to the family Solanaceae. A number of important crops—corn, soybean, sunflower, and others—have been found to contain accidental impurities caused by the parts of the jimsonweed plant [30]. 

The most widespread source of accidental TAs consumption in agriculture is the contamination of crops by plants from the Solanaceae family. There are three specific species responsible for the contamination of food and feed products by TAs, and these are Datura, Hyoscyamus, and Atropa [25]. As a result of their easy growth, they have the potential to become weeds in crops of various food plants. Additionally, these alkaloids are present in all parts of plants that produce TAs, resulting in frequent cross-contamination, especially with seeds as well as leaves, roots, fruits, and flowers. As a result of its wide distribution, the seeds of jimsonweed are commonly found in all warm regions of the world, along with other Datura species such as the long spined thorn apple (*Datura ferox*), which is commonly consumed in food.

Many crops can be contaminated with TAs, such as buckwheat, maize, millet, soybean, sunflower, and wheat [31,32]. Along with the ingestion of the contaminated food, humans can be exposed to the TAs when ingesting edible plants which are mixed up with the TAs containing plant parts [13]. In 2015, Recommendation 2015/976/EU1 in monitoring the TAs presence in food was adopted [33]. In 2016, the maximum atropine and scopolamine levels were limited to 1μg/kg for each alkaloid in cereal-based food for young children and infants [15].

The main objective of this paper is to offer information about the occurrence of TAs in more than 100 samples of maize products collected from the retail stores in the Republic of Serbia during 2021 (corn grits, polenta, and semolina), with a special interest in atropine and scopolamine, to emphasize a phenomenon of international significance.

## 2. Results

The presence of investigated toxins was determined in 33 corn grit samples. According to Table 1, in 11 samples, both atropine and scopolamine were detected, 2 samples contained only atropine, while the rest of the samples (60.6%) were below the LOQ. Atropine to scopolamine ratio was in the scale of 2.0 to 4.0. 

Out of the 39 tested samples of polenta, 82.1% of the samples were below the LOQ, 4 had the detections of both toxins, while in 3 samples, only atropine was detected. The atropine: scopolamine ratio ranged from 0.4 to 3.7 (Table 1).

In the case of the studies, regarding semolina samples (31 in total), 8 samples contained both atropine and scopolamine and in 4 samples only atropine was detected, while 61.3% of the samples were below the LOQ. The ratio between the toxins, in this case, was in the range of 1.8–5.8 (Table 1).

From the results showed in Table 1, the same distribution of atropine (semolina > corn grits > polenta) and scopolamine (semolina > corn grits > polenta) can be seen, respectively. Furthermore, the obtained results have shown that polenta samples had the lowest sum of atropine and scopolamine (2.17 μg/kg), while the highest sum of atropine and scopolamine was noted for semolina samples (69.00 μg/kg). In all of the studied samples, great co-occurrence of the two tested toxins was noted. The lowest atropine concentration (1.10 μg/kg) was detected in a polenta sample, while the highest concentration was observed in a semolina sample (58.80 μg/kg). In the case of scopolamine, a sample of polenta had the lowest concentration (1.07 μg/kg), while the highest concentration was detected, just like in the case of atropine, in a sample of semolina (10.20 μg/kg) (Table 1).

**Figure 1:**
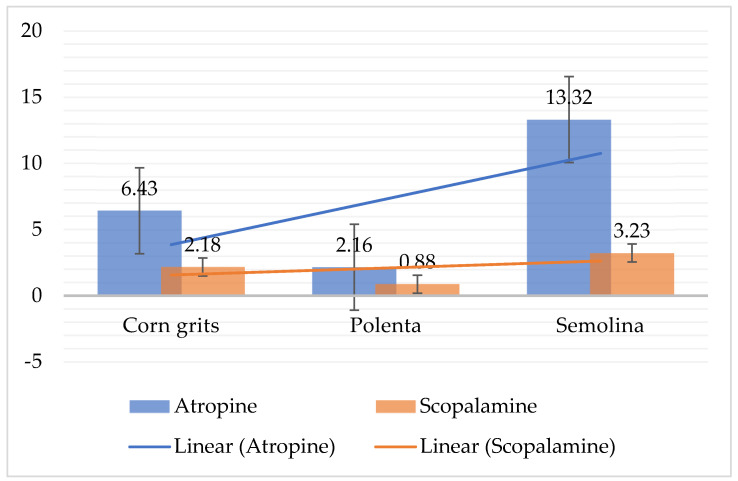
Atropine and scopolamine relationship in investigated samples, µg/kg.

Concerning Figure 1, in corn grits, the ratio between atropine and scopolamine concentrations in the detected samples was 2.95, in polenta 2.45, and in semolina was 4. This means that in the positive samples, the atropine concentrations were 2.5 to 4 times higher than scopolamine detections. However, in the samples where the atropine concentration was low or just above LOQ, the detections of scopolamine were below the limit of quantification. 

**Figure 2:**
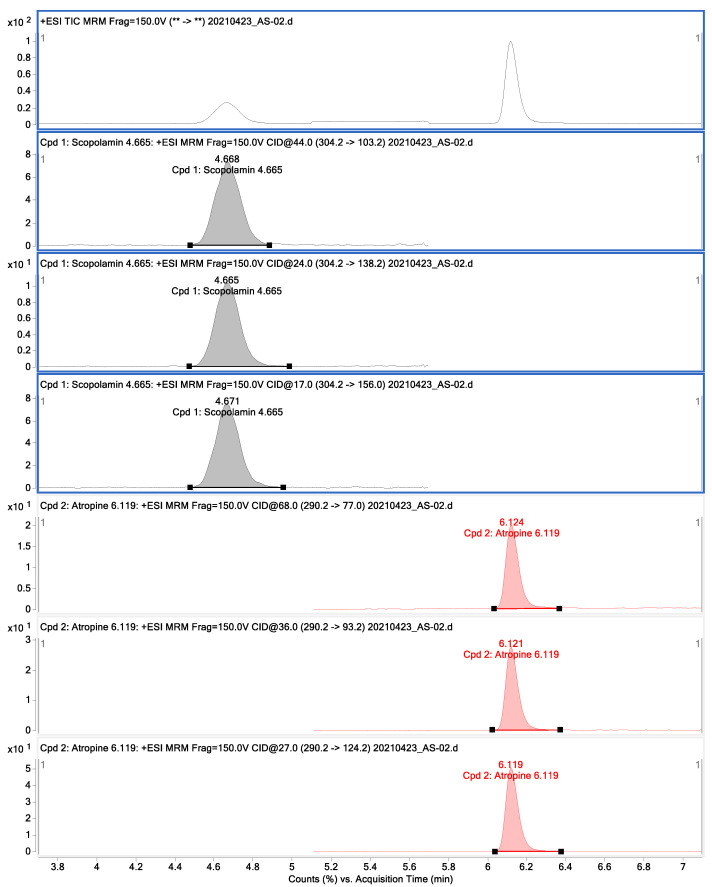
The chromatograms of atropine and scopolamine obtained by the LC-MS/MS.

The chromatograms of atropine and scopolamine obtained by the LC-MS/MS are shown in Figure 2.

## 3. Discussion

To this day, studies conducted in Europe showed that the TAs can be found as contaminants in various types of food commodities, such as teas, flours, honey, and infant formulas, while the most common TAs detected in cereal-based foods were concluded to be atropine and scopolamine [33,34]. 

The study of the 18 corn puff samples showed that 22.2% of all the samples had atropine (1.03–1.58 μg/kg) and scopolamine (0.29–0.47 μg/kg) detections [15]. Out of the 12 tested popcorn samples in 41.7% atropine (from 5.3 to 28.0 μg/kg) and scopolamine (from 2.1 to 6.3 μg/kg) were detected.

The testing of 113 cereal-based products for young children and infants between 2011 and 2014 in the Netherlands showed that 25 samples had atropine and scopolamine detections. The contaminated samples were exclusively the samples of the cereals which are supposed to be mixed with the milk. The highest detected concentrations were 65.6 μg/kg for atropine and 15.2 μg/kg for scopolamine [34].

When 15 samples of the gluten-free grains and flours were tested, in 60% of the samples at least one of the TAs was detected. The atropine concentrations ranged from 7 ± 1 to 78 ± 12 µg/kg, while the range for scopolamine was from 28 ± 6 µg/kg [35].

According to the RASFF (Rapid Alert System for Food and Feed) [36] from January 2020 to this day, there were 19 reports considering the contamination by the TAs, out of which 14 were marked as a serious risk, in 4 cases no decision has been made yet, while only 1 was characterized as nonserious. The cases marked as serious include TAs detections in corn tortilla chips, organic tortilla chips, savory, soybean meal, organic corn flour, organic flaxseed meal, buckwheat flour, parsley, millet, deep frozen spinach puree, popcorn maize, organic blackberry leaves, and organic soy flakes. The highest TAs concentrations were reported for deep frozen spinach puree from Slovakia (atropine: 850.0–3446.0 μg/kg, scopolamine: 1033.0–3860.0 μg/kg), while the ranges in case of the other serious reports were from 19.0 to 543.1 μg/kg for atropine and from 1.2 to 87 ± 35 μg/kg for scopolamine. 

Between 1994 and 2020 there were 6 cases of corn and corn-based products contaminated by the TAs, with the sum of atropine and scopolamine ranging from 8.4 to 252.0 μg/kg [37]. 

If we compare the results obtained in this research with the lowest atropine and scopolamine concentrations in the mentioned serious cases, it can be concluded that at least 17 out of the total 103 tested samples, i.e., 16.5%, could be marked as a serious threat by the RASFF.

According to the scientific literature, the concentrations of atropine and scopolamine above the LOQ pose a potential hazard to human and animal health, with the most affected being infants, children, and people with heart conditions [37,38,39,40]. Taking into account the previously stated, the results of this study may be considered alarming, since 31.1% of the tested samples were contaminated with atropine and scopolamine in concentrations above the LOQ.

Some of the most recent indications of how dangerous TAs in food may be are the food aid outbreak in Uganda three years ago [37] and the study conducted on dairy cows which was published last year [41].

The food aid outbreak occurred due to the food contamination with the TAs because of the presence of *D. stramonium* L. seeds. Namely, after ingesting the TAs-contaminated “Super Cereal” (cereals and soybean blend), 315 people got sick, while 5 of them died [37]. 

The study by Lamp et al. [41] pointed out the fact that we are still not aware of all the food products that could potentially be a health hazard due to contamination with the TAs. In other words, their research is presenting the transfer of the TAs from the feed into the cow’s milk for the first time. The authors also stated that 279 μg/kg of body weight is the highest subclinical dose possible. 

## 4. Conclusions

The highest concentrations of the studied TAs were observed in a semolina sample (atropine: 58.80 μg/kg, scopolamine: (10.20 μg/kg)). Out of the total 103 tested samples, 32 samples (31.1%) were contaminated with atropine and scopolamine in concentrations above the LOQ.

The results of this study, since the TAs concentrations above the LOQ are considered potential human and animal health hazards, are pointing out the need for large-scale continuous monitoring of raw materials and food products meant for human and animal diets.

## 5. Materials and Methods

### 5.1. Chemicals and Reagents

Atropine and scopolamine reference standards were obtained from Sigma-Aldrich. The standard solutions of atropine and scopolamine were prepared at 1 mg/mL in methanol, each. The working standard solution mixtures were prepared at 10 µg/mL and 1 µg/mL in methanol and stored in the dark at −20 °C. Acetonitrile and methanol were purchased from J.T. Baker. Both organic solvents were HPLC Ultra Gradient HPLC grade. The formic acid was analytical grade (Fisher Scientific, Loughborough, UK). The Hillium QuEChERS extraction pouch 550 mL (P/N QEHLL0510P, Agilent Technologies, Santa Clara, USA) and Hillium QuEChERS dispersive kit 15 mL (P/N QDHLL15032, Agilent Technologies, Santa Clara, USA) were used for the extraction and clean-up [42].

### 5.2. Instrumentation

HPLC Agilent 1290 Infinity II chromatograph equipped with a quaternary pump, multisampler, and column compartment thermostat was used for the detection of atropine and scopolamine. The HPLC system was coupled to an Agilent 6495 LC/TQ triple quadrupole mass spectrometer with AJS ESI (Jet Stream Technology Ion Source). The Zorbax Eclipse Plus C18 column Rapid Resolution HD (50 × 2.1 mm, 1.8 µm particle size) was used for the chromatographic separation. The column temperature was held at 35 °C and the injection volume for the LC system was 2 µL. The chromatographic separation of AT and SC was carried out with a mobile phase consisting of water (A) and methanol (B), both containing formic acid (0.1%, *v*/*v*), in a gradient mode and flow rate of 0.25 mL/min. A gradient elution started at 5% of B and held for 1 min. This composition was increased to 40% B at 7 min, 90% B at 8 min, and held for 2 min. The composition of the mobile phase returned to the initial conditions in 1 min and the system was equilibrated for 2 min. The total running time was 11 min. The ESI source was used with the following settings: drying gas (nitrogen) temperature of 200 °C, drying gas flow rate 16 L/min, nebulizer pressure 30 psi, sheath gas temperature of 300 °C, sheath gas flow 12 L/min, and capillary voltage 3000 V. The detection was performed using the dynamic multiple reactions monitoring mode (dMRM). The Agilent MassHunter software (v.B.10.0 SR1 Agilent Technologies, 2006–2019, Santa Clara, United States) was used for the optimization and quantification [15].

### 5.3. Sample Collection and Preparation

A total of 103 samples of maize products were analyzed for the presence of toxins, i.e., TAs. The samples were collected from the retail stores in the Republic of Serbia in 2021 and analyzed for the presence of atropine and scopolamine (33 corn grits, 39 polenta, and 31 semolina samples). The sampling was performed following the EU directive 2002/63/EC [43]. The samples were ground into a powder before the analysis.

**Figure 3:**
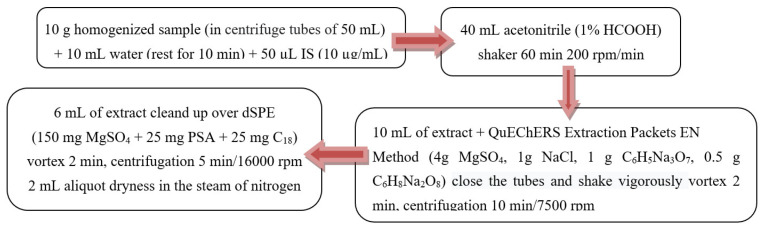
The steps of the atropine and scopolamine extraction.

Atropine and scopolamine were extracted from ground corn puff samples using the QuEChERS method described in Figure 3.

### 5.4. Validation Parameters

Atropine and scopolamine were analyzed using ESI+ (electrospray positive ionization) by dynamic multiple reactions monitoring mode. The fragmentation of the protonated atropine and scopolamine ions yielded three product ions, respectively (Table 2). The most intense MRM transitions for atropine *m*/*z* 290.2 > 124.2 and scopolamine 304.2 > 138.2 were monitored for quantification and the second most intense (other three) transitions were used for the confirmation [15]. 

The study conducted by Vuković et al. [44] pointed out that the addition of formic acid to the mobile phase improved ionization efficiency and gave the studied tropane alkaloids a finer peak. The multiple reaction monitoring chromatograms and mass spectra of investigated TAs transitions are given in Figure 2. The retention time (Rt) of atropine was 9.63 min, while the Rt of scopolamine was 8.42 min.

The limit of detection (LOD) was based on the signal-to-noise ratio, calculated by MassHunter software at 0.1 μg/kg, whereas the limit of quantification (LOQ) was set at 1 μg/kg, for both atropine and scopolamine in all the matrices, which was following the Commission Recommendation (EN) 2015/976 [45] on the monitoring of the presence of tropane alkaloids in food-related to the LOQ.

In this study, tropane alkaloids standards were spiked into different matrices at five calibration levels of 1, 2, 5, 10, and 20 g/kg. Atropine and scopolamine concentrations in samples were calculated using the obtained calibration curves (both atropine and scopolamine) [15]. The coefficient correlation (R^2^) with the obtained average recoveries (Rec ± RSD, %) for each matrix were shown in Table 3.

## Figures and Tables

**Table 1:** Occurrence of atropine and scopolamine in the studied corn grits, polenta, and semolina samples.

	Atropine	Scopolamine	Sum of Atropine and Scopolamine
**Corn grits**			
N	33	33	33
N pos (%)	13 (39.4)	11 (33.3)	13 (39.4)
min c (μg/kg)	2.28	1.12	3.40
max c (μg/kg)	16.33	6.16	22.49
SD	3.8	1.5	
SE	1.1	0.4	
Median	5.3	2.1	
Q1	4.2	1.3	
Q3	7.2	2.5	
**Polenta**			
N	39	39	39
N pos (%)	7 (17.9)	4 (10.3)	7 (17.9)
min c (μg/kg)	1.10	1.07	2.17
max c (μg/kg)	3.98	2.80	6.78
SD	1.1	1.0	
SE	0.4	0.4	
Median	2.2	1.1	
Q1	1.2	0.0	
Q3	2.7	1.1	
**Semolina**			
N	31	31	31
N pos (%)	12 (38.7)	8 (25.8)	12 (38.7)
min c (μg/kg)	1.20	1.10	2.30
max c (μg/kg)	58.80	10.20	69.00
SD	18.2	3.8	
SE	5.3	1.1	
Median	5.7	1.4	
Q1	2.2	0.7	
Q3	11.8	5.4	

N—number of the samples; pos—positive samples; c—concentration; SD—standard deviation; SE—standard error; Q1—First quartile 25% percentile; Q3—Third quartile 75% percentile; LOD = 0.1 µg/kg; LOQ = 1.0 µg/kg for atropine and scopolamine.

**Table 2:** Liquid chromatography electrospray ionization tandem mass spectrometric parameters for the analysis of atropine and scopolamine in multiple reaction monitoring mode.

TA	Molecular Formula	Molecular Weight (g/mol)	Retention Time (min)	Precursor Ion [M+H *] (*m*/*z*)	Product Ion (*m*/*z*)	Fragmentation Voltage (V)	Collision Energy (V)
AT	C_17_H_23_NO_3_	289.2	9.63	290.2	124.2 *	166	24
					93.2	166	36
					77.1	166	68
SC	C_17_H_21_NO_4_	303.2	8.42	304.2	156	166	12
					138.2 *	166	24
					103.2	166	44

*—Quantification product ion.

**Table 3:** Linearity and recovery.

Matrix	Corn Grits	Polenta	Semolina
R^2^	0.9974	0.9987	0.9963
Rec, %	86.8 ± 14.6	89.1 ± 16.1	85.3 ± 15.8

## Data Availability

Data is contained within the article.
